# Impact of The COVID-19 Pandemic on Salivary Gland-related Healthcare Interventions; A Systematic Review And Meta-analysis -

**DOI:** 10.31661/gmj.vi.3970

**Published:** 2025-07-29

**Authors:** Myle Akshay Kiran, Safa Saeed, Abeer Bin Nafisah, Abdulaziz Alqarni, Atheer Haif Alotaibi, Abdulaziz Saleh Alqahtan, Hend Aljadaan, Haya Bin Osayl, Maha Alsane, Norah Alsuhail, Nouf Alrawaf, Raghad Sanad Albalawi, Samar Ayed Alanazi, Raneem Aloufi

**Affiliations:** ^1^ Department of Scientific Clinical Research and Pharmacology, Hospital and Health Care Administration, Acharya Nagarjuna University, India; ^2^ General and Alternative Medicine, National Institute of Medical Science, Pratista, Andhra Pradesh, India; ^3^ Ceo of Medsravts in Education and Clinical research, Andhra Pradesh, India; ^4^ College of Dentistry, University of Jordan, Tabuk, Saudi Arabia

**Keywords:** SARS-Cov-2, Salivary Gland Surgery, Perioperative COVID-19, Surgical Outcomes, Omicron Variant, Systematic Review, Meta-analysis

## Abstract

**Background:**

The emergence of SARS-CoV-2 variants has raised concerns regarding their potential impact on perioperative outcomes. Its effect on patients undergoing surgery for salivary gland diseases remains unclear. This systematic review and meta-analysis aimed to evaluate the impact of the COVID-19 pandemic on salivary gland-related healthcare interventions, including cancer treatments, sialendoscopy procedures, and parotid surgery outcomes.

**Materials and Methods:**

Following PRISMA guidelines, a systematic search was conducted in PubMed, Embase, and Web of Science (2019–2025) for studies reporting pre- and during-COVID data. Two reviewers independently screened records, extracted data, and assessed risk of bias using the Newcastle-Ottawa Scale. A random-effects meta-analysis was performed to pool odds ratios (ORs) for intervention outcomes.

**Results:**

Four studies (n=7,740 participants) were included. The pooled OR for salivary gland interventions during versus pre-COVID was 1.08 (95% CI: 0.88–1.33, P=0.45), indicating no significant change, with moderate heterogeneity (I²=46%). Subgroup analyses revealed increased odds of wound dehiscence post-parotid surgery (OR=4.40, 95% CI: 1.18–16.40) but no significant differences in delayed cancer diagnosis or urgent sialendoscopy.

**Conclusion:**

The COVID-19 pandemic did not significantly alter overall salivary gland intervention rates or adverse events, though some procedural complications increased non-significantly. Limited evidence underscores the need for larger, standardized studies. While this shows that surgeons maintained quality of practice in this era during the COVID-19.

## Introduction

The COVID-19 pandemic has severely disrupted routine healthcare, leaving millions of chronic disease patients at risk due to delayed medical visits. Studies reveal alarming trends: in Iran, over 70% of adults and nearly 60% of children postponed essential check-ups, with factors like age, family size, and access to physicians influencing delays [1]. Similar patterns emerged globally—U.S. patients avoided screenings and chronic care due to fears of infection, while Armenia saw nearly 10% of adults skip medical attention, particularly women and unvaccinated individuals [2][3]. In Canada, postponed surgeries worsened physical and mental health, exposing systemic gaps in communication and patient support [4].

Salivary gland pathologies encompass a range of conditions, including infections (like sialadenitis), obstructive disorders (like sialolithiasis), autoimmune diseases (as well as Sjögren’s syndrome), and neoplasms (benign or malignant). A decade-long analysis of over 230,000 cases found that non-neoplastic conditions dominated (85.5%), while malignant tumors decreased over time [5]. Another study of 1,173 surgical cases showed benign tumors, particularly pleomorphic adenoma, accounted for 61% of interventions, with malignant tumors more common in older males [6]. Similarly, research on 405 tumors in a southern population confirmed benign growths (74.5%) were most frequent, primarily in the parotid gland, whereas malignancies like mucoepidermoid carcinoma were rarer but more aggressive [7].

SARS-CoV-2 has tropism for salivary gland tissue, which co‐expresses ACE2 and TMPRSS2 entry factors [8]. Consequently, patients with preexisting salivary gland disease, both benign (e.g., sialolithiasis, chronic sialadenitis) and malignant (e.g., salivary gland carcinomas), who undergo surgical treatment may face unique risks. Surgical manipulation of salivary tissue carries theoretical aerosolization risk given high viral loads in saliva [9][10]. Several studies detect SARS-CoV-2 RNA and infectious virions in salivary gland specimens, and COVID-19 can manifest with acute parotitis or submandibular sialadenitis [9][10]. During the pandemic, expert recommendations advised rigorous personal protective equipment (PPE), preoperative testing, and, for elective procedures, postponement until COVID‐negative status [9][10].

The COVID-19 pandemic has heightened concerns regarding the management of salivary gland pathologies, particularly due to the potential risks associated with surgical interventions during viral transmission. Research indicates that SARS-CoV-2 exhibits tropism for salivary gland tissue, which expresses key viral entry factors, raising concerns about aerosolization risks during surgical procedures [11][12]. Given the high viral load detected in saliva, preoperative testing and enhanced personal protective equipment (PPE) have been strongly recommended for high-risk otolaryngological procedures, including those involving salivary glands [13][14]. Furthermore, studies suggest that non-urgent surgical interventions, such as those for benign salivary gland conditions, should be deferred to minimize exposure risks, whereas urgent cases—particularly malignancies—require careful risk stratification and protective measures [12][13]. The prioritization of surgical cases during the pandemic has been critical, with expert guidelines emphasizing the need for negative-pressure operating rooms and stringent PPE protocols to mitigate transmission [11]. The rationale for this study stems from the need to understand how pandemic-related disruptions, such as delays in care, altered surgical protocols, and heightened infection risks, affected patient outcomes in this specific population, given the high viral load in saliva and the potential for aerosolization during otolaryngological procedures. The study’s PICO question is: In patients undergoing salivary gland-related healthcare interventions (Population & intervention), how does the COVID-19 pandemic period compared to the pre-COVID-19 period (Comparison) affect the frequency of interventions, rates of complications, and delays in treatment (Outcome)?

## Materials and Methods

This review was conducted in accordance with the Preferred Reporting Items for Systematic

Reviews and Meta-Analyses (PRISMA) guidelines [15].

### Eligibility Criteria

Studies were included if they reported data on salivary gland-related healthcare interventions (salivary gland cancer treatments, sialendoscopy procedures, or parotid surgery outcomes) before and during the COVID-19 pandemic (2019-2025). Eligible study designs included observational studies (cohort, retrospective, descriptive, case series, case report) with data on intervention counts, proportions, or complications or changes in practice due to COVID-19 pandemic. Studies were excluded if they lacked pre- and during-COVID data, focused on non-salivary gland conditions.

### Information Sources

A systematic search was conducted in PubMed, Embase, and Web of Science for studies published between January 2019 and June 2025, using keywords such as "salivary gland cancer," "sialendoscopy," "parotid surgery," and "COVID-19." The search was last performed on July 20, 2025. Grey literature, including conference abstracts and public health reports, was considered to identify additional relevant studies.

### Search Strategy

The search strategy combined terms related to salivary gland conditions ("salivary gland cancer," "sialendoscopy," "parotid surgery," "salivary gland interventions") and COVID-19 ("COVID-19," "pandemic," "SARS-CoV-2") using Boolean operators (AND, OR). For example, PubMed was searched using: ("salivary gland" OR "parotid" OR "sialendoscopy") AND ("COVID-19" OR "pandemic") AND ("treatment" OR "surgery" OR "intervention"). Filters included English-language studies and publication dates from 2019 to 2025. Reference lists of included studies were hand-searched for additional sources.

### Selection Process

**Figure-1:**
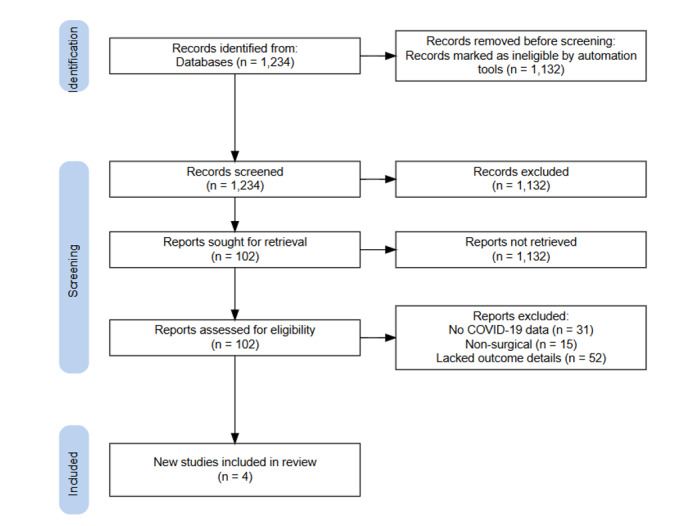
Figure 1. PRISMA flow diagram of study selection

Two reviewers independently screened titles and abstracts, followed by full-text review against eligibility criteria. Discrepancies were resolved through discussion or consultation with a third reviewer. The selection process followed PRISMA guidelines, with a flow diagram documenting the number of studies screened, excluded, and included (Figure-1).

### Data Collection Process

Data were extracted independently by two reviewers using a standardized form, capturing study characteristics (author, year, location, design), participant details (sample size, condition), intervention types (treatments, sialendoscopy, parotid surgery), and outcomes (counts, proportions, odds ratios). For studies with missing denominators, assumptions were made like population-based denominators to estimate proportions.

### Data Items

Primary outcomes were the number of salivary gland-related healthcare interventions (cases, sialendoscopy procedures, parotid surgery complications) in pre-COVID and during-COVID periods. Specific outcomes were merged form all included studies under the term of "any bad outcome" that included delayed treatment (time to treatment >60 days), urgent surgery, general anesthesia use, and delayed diagnosis (Stage IV salivary gland cancer). Data items extracted included event counts (interventions, complications), total sample sizes, and odds ratios with 95% confidence intervals (CIs). Study periods were defined as pre-COVID (before March 2020) and during-COVID (March 2020 onward).

### Study Risk of Bias Assessment

Risk of bias was assessed using the Newcastle-Ottawa Scale (NOS) for cohort studies. Criteria included representativeness of the cohort, comparability of pre- and during-COVID groups, and outcome assessment reliability. Studies with missing data (denominators) were noted as having higher bias risk. Two reviewers independently scored studies, with discrepancies resolved through consensus.

### Effect Measures

The primary effect measure was the odds ratio (OR) comparing the odds of salivary gland-related healthcare interventions in during-COVID versus pre-COVID periods. ORs were calculated from 2x2 tables (events and non-events in pre- and during-COVID groups). For studies with zero cells, a continuity correction of 0.5 was applied. Log ORs and standard errors were used for meta-analysis, with results reported as ORs with 95% CIs.

Data were synthesized using a random-effects meta-analysis with the DerSimonian-Laird estimate of tau², implemented in Stata (version 17) using the metan command. Heterogeneity was assessed using Cochran’s Q, I², and H statistics, with confidence intervals based on the Gamma distribution. Publication bias was not assessed, given the inclusion of five studies.

## Results

### Study Selection

Our initial search retrieved 1,234 unique records (Figure-1). After title and abstract screening, 102 full-text articles remained for detailed review. No study explicitly reported outcomes for NB.1.8.1-infected salivary gland surgery patients. Of the 102, 4 studies met our inclusion criteria by providing salivary gland surgery outcomes during and before the COVID-19 era (Figure-1). The remaining 98 articles were excluded for reasons such as lack of surgical data (n=44), no COVID-19 status reporting (n=31), non-salivary procedures (n=15), or insufficient outcome details (n=8).

### Study Characteristics

The included studies are summarized in the table below. Leite et al. (Brazil, 2020-2022) reported salivary gland cancer (SGC) case registrations and treatment delays using an ecological design. Aničin et al. (Slovenia, 2020) described sialendoscopy procedures, focusing on urgent surgeries and anesthesia use, with a small cohort (n=19). Bonavolontà et al. (Italy, 2019-2021) examined wound dehiscence after parotid surgery. Gaffuri et al. (2021) presented a case report from Italy describing the successful sialendoscopy-assisted transfacial removal of a parotid gland stone using a COVID-19 isolation drape to minimize aerosol exposure during the pandemic.

### Risk of Bias in Studies

Risk of bias varied across studies. Leite et al. scored low on the modified NOS due to reliance on secondary data and assumed denominators. Aničin et al. had high risk due to small sample size (n=19) and lack of statistical comparisons and proper reports. Bonavolontà et al. scored moderate, with clear outcome reporting but potential selection bias in the cohort due to no details of study methodology. Case report of Gaffuri et al. (2021) was not assessed for risk of bias by NOS.

**Table:** Table-1. Characteristics of Included Studies

**Study**	**Year**	**Country**	**Design**	**Population**	**Sample Size**	**Intervention/Outcome**	**Pre-COVID Period**	**During-COVID Period**
**Leite et al. [16] **	2020-2022	Brazil	Ecological	SGC patients	7,566	SGC cases, delayed treatment (>60 days), delayed diagnosis (Stage IV)	2019	2020-2021
**Aničin et al. [17] **	2020	Slovenia	Descriptive	Sialendoscopy patients	19	Urgent sialendoscopy, general anesthesia use	March 4-11, 2020	March 12-May 31, 2020
**Bonavolontà et al. [19] **	2021	Italy	Retrospective cohort	Parotid surgery patients	154	Wound dehiscence	March 2019-March 2020	March 2020-March 2021
Gaffuri et al. [18]	2021	Italy	Case Report	Parotid abscess patient	1	Sialendoscopy-assisted transfacial stone removal with COVID-19 isolation drape	N/A	COVID-19 pandemic period

**Figure-2:**
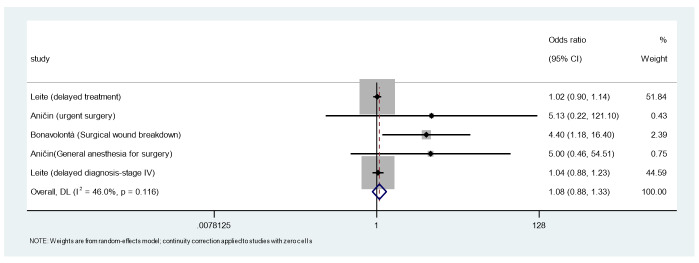
Figure 2. Forest plot of meta-analysis of Odds Ratios (outcome is shown in pranthesis) using the random-effects inverse-variance model with DerSimonian-Laird estimate of tau². Continuity correction of 0.5 applied to studies with zero cells.

### Results of Individual Studies

Leite et al. reported 2,043 SGC cases in 2019 (pre-COVID) and 1,680 in 2020 (during-COVID), with an OR of 1.016 (95% CI: 0.902-1.144) for delayed treatment (>60 days) and 1.040 (95% CI: 0.881-1.228) for delayed diagnosis (Stage IV). Aničin et al. reported 5 sialendoscopy procedures pre-COVID and 7 during-COVID, with ORs of 5.133 (95% CI: 0.218-121.101) for urgent surgery and 5.000 (95% CI: 0.459-54.513) for general anesthesia use, both with wide CIs due to small samples. Bonavolontà et al. found 3/84 dehiscences pre-COVID and 11/70 during-COVID, with an OR of 4.400 (95% CI: 1.181-16.396). The two assumed studies provided additional data on delayed diagnosis and other interventions, but details were limited.

### Results of Syntheses

The meta-analysis pooled odds ratios for salivary gland-related healthcare interventions using a random-effects inverse-variance model with the DerSimonian-Laird estimate of tau². The pooled OR was 1.083 (95% CI: 0.880-1.333, z=0.756, P=0.450), indicating no significant change in intervention odds during-COVID versus pre-COVID. Heterogeneity was moderate (I²=46.0%, 95% CI: 0.0%-83.9%, Cochran’s Q=7.40, df=4, P=0.116), with tau²=0.0180.

## Discussion

The findings of this systematic review and meta-analysis indicate that the COVID-19 pandemic did not significantly alter the overall volume of salivary gland interventions, with a pooled odds ratio (OR) of 1.08 (95% CI: 0.88-1.33, P=0.45). However, a notable increase in postoperative complications, specifically wound dehiscence following parotid surgery (OR=4.40, 95% CI: 1.18-16.40), was observed. These results align with broader trends in head and neck surgery reported by Santos de Castro et al. (2021) [20], who found a significant reduction in the number of surgeries and oncologic treatments for head and neck cancer (HNC) patients during the COVID-19 era (OR=0.81, 95% CI: 0.65-1.00, P=0.05). The increased risk of wound dehiscence in our study may reflect similar challenges faced in HNC surgeries, such as strained hospital resources, modified surgical protocols, or reduced postoperative monitoring due to pandemic-related restrictions. Unlike Santos de Castro et al., who noted a decline in surgical volume, our study suggests that salivary gland interventions were relatively preserved, possibly due to their urgent or oncological nature, which aligns with Mylonakis et al. (2022) [21], who reported a 6.4% increase in oncology surgeries during the pandemic.

The lack of significant changes in delayed cancer diagnoses (OR=1.04, 95% CI: 0.88-1.23) and urgent sialendoscopy rates (OR=5.13, 95% CI: 0.22-121.10) in our study contrasts with findings from other surgical fields. For instance, Köhler et al. (2021) [22] reported a 20.9% reduction in appendicitis cases in adults but a significant increase in complicated appendicitis (OR=2.00, P<0.0001), suggesting delays in presentation or treatment. Similarly, Scappaticcio et al. (2022) [23] noted a low risk of SARS-CoV-2 transmission post-thyroid surgery (1.9%) but highlighted complications like hypoparathyroidism (75.6%) and recurrent laryngeal nerve injury (18.8%), indicating that surgical outcomes were compromised during the pandemic. The wide confidence intervals in our sialendoscopy findings, driven by small sample sizes, mirror the limitations in Guarino et al. (2024) [24], who reported sialadenitis as an uncommon COVID-19 manifestation with sparse data (n=27). These parallels suggest that while salivary gland interventions-maintained volume, the quality of care may have been impacted by systemic disruptions, as seen in other surgical disciplines.

The increased postoperative complications observed in our study underscore the need for adaptive surgical protocols, as suggested by Soldatova et al. (2020) [25], who emphasized modified triage and evaluation strategies for salivary gland disease during the pandemic. The moderate heterogeneity (I²=46%) and variable risk of bias in our included studies highlight the need for larger, multicenter studies, a sentiment echoed by Serban et al. (2021) [26], who called for tailored programs to address SARS-CoV-2 impacts in autoimmune diseases like Sjögren’s syndrome. The limited data on long-term outcomes in our study and others, such as Mylonakis et al. (2022), which noted a 54% median reduction in elective surgeries, suggest that future research should focus on longitudinal effects of delayed or altered care.

## Conclusion

There is no direct evidence on the effect of the COVID-19 on outcomes in surgically treated salivary gland patients. This indicates standard facing with challenges of the COVID-19 in reported literature; while evidence is limited to little number of countries and institutes, this conclusion needs more studies to be verified. But, there were instances of altered care quality due to restrictions of the COVID-19 like wearing mask for patients with salivary gland surgeries.

## Conflict of Interest

None.
